# What Are Sheep Doing? Tri-Axial Accelerometer Sensor Data Identify the Diel Activity Pattern of Ewe Lambs on Pasture

**DOI:** 10.3390/s21206816

**Published:** 2021-10-13

**Authors:** Seer J. Ikurior, Nelly Marquetoux, Stephan T. Leu, Rene A. Corner-Thomas, Ian Scott, William E. Pomroy

**Affiliations:** 1 School of Veterinary Science, Massey University, Palmerston North 4410, New Zealand; R.Corner@massey.ac.nz (R.A.C.-T.); I.Scott@massey.ac.nz (I.S.); W.Pomroy@massey.ac.nz (W.E.P.); 2 College of Veterinary Medicine, University of Agriculture, Makurdi 970231, Nigeria; 3 EpiCentre, School of Veterinary Science, Massey University, Palmerston North 4410, New Zealand; N.Marquetoux@massey.ac.nz; 4 School of Animal and Veterinary Sciences, University of Adelaide, Roseworthy 5371, Australia; stephan.leu@adelaide.edu.au; 5 School of Agriculture and Environment, Massey University, Palmerston North 4410, New Zealand

**Keywords:** sheep, diel activity, classification algorithm, tri-axial accelerometers, health monitoring

## Abstract

Monitoring activity patterns of animals offers the opportunity to assess individual health and welfare in support of precision livestock farming. The purpose of this study was to use a triaxial accelerometer sensor to determine the diel activity of sheep on pasture. Six Perendale ewe lambs, each fitted with a neck collar mounting a triaxial accelerometer, were filmed during targeted periods of sheep activities: grazing, lying, walking, and standing. The corresponding acceleration data were fitted using a Random Forest algorithm to classify activity (=classifier). This classifier was then applied to accelerometer data from an additional 10 ewe lambs to determine their activity budgets. Each of these was fitted with a neck collar mounting an accelerometer as well as two additional accelerometers placed on a head halter and a body harness over the shoulders of the animal. These were monitored continuously for three days. A classification accuracy of 89.6% was achieved for the grazing, walking and resting activities (i.e., a new class combining lying and standing activity). Triaxial accelerometer data showed that sheep spent 64% (95% CI 55% to 74%) of daylight time grazing, with grazing at night reduced to 14% (95% CI 8% to 20%). Similar activity budgets were achieved from the halter mounted sensors, but not those on a body harness. These results are consistent with previous studies directly observing daily activity of pasture-based sheep and can be applied in a variety of contexts to investigate animal health and welfare metrics e.g., to better understand the impact that young sheep can suffer when carrying even modest burdens of parasitic nematodes.

## 1. Introduction

Monitoring the activity of animals using non-invasive technologies such as accelerometer sensors can provide an indicator of an individual’s response to its external or internal environment [1]. These technologies are widely used in wildlife ecology studies [2,3,4,5] and in an increasing number of animal science studies [6,7,8,9,10,11,12,13] to infer the behavioural responses of host animals to their environment.

In domestic sheep, accelerometer data has been used to develop classifier algorithms to identify different activities of several breeds of sheep on pasture, including Merino [6,9,14,15,16,17], Sarda [18], Suffolk cross [19] and Debouillet [20]. The range of activity types include grazing, lying down, standing, walking, and ruminating. A few studies have endeavoured to use such activity classifiers to investigate behavioural patterns, usually associated with a particular context, e.g., to detect the effect of opioids on sheep behaviour [21], to monitor change in behaviour during parturition events [20,22], and detection of lameness [23,24]. In the field of parasitology, helminth worms have been associated with daily changes in sheep lying time [25] and increased irregularity in activity patterns [26], as measured by accelerometers. The current authors found that sub-clinically parasitised sheep reduced their overall activity compared to uninfected controls [27] using an ultra-light weight acceleration sensor (Actigraph wGT3X-BT^®^, LLC, Pensacola, FL, USA). This commercially available sensor has been used in proximity studies investigating contact between ewes and their lambs [28], and can potentially assist in the identification of different activity types in pastured sheep in order to allow onward investigations on the influence of parasites on these behavioural attributes. Each species differs in their signature in the tri-axial data, and hence the developments made in developing classifier algorithms in other species using the Actigraph wGT3X-BT^®^ on horses [29] and dogs [30] cannot be applied to sheep. Moreover, the ability to detect changes in animal activity during a 24 h period is potentially useful for detecting health and welfare problems, but few studies have used accelerometry data to identify the diel activity of sheep. One study to date has used a motion index derived from accelerometer data to investigate the circadian rhythm of sheep [31], but the effect of time of day on activity classes such as grazing, standing, lying and walking have received little attention from a research standpoint.

**Figure 1:**
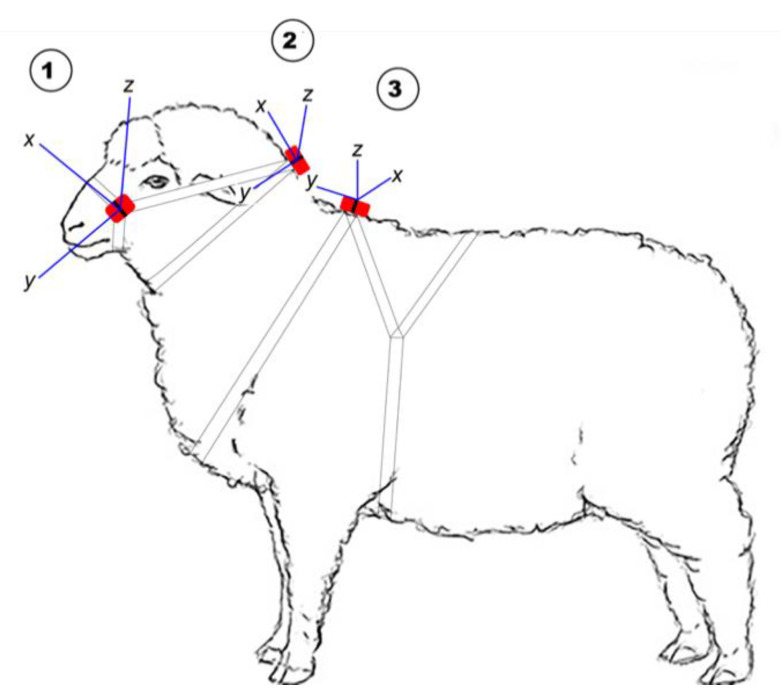
The position of the Actigraph tri-axial accelerometer on 1 (head halter), 2 (neck collar) and 3 (body harness) and the axis orientation at each position in relation to the animal’s body.

For the limited studies reporting simultaneous deployment of accelerometers using different attachment locations in sheep, accelerometers have been applied to individuals on the hind leg [32], foreleg [14], neck [14,18,19,33] and ear [14,19,33], and each may have advantages. It has been suggested that for an accurate indication of individual energy expenditure, accelerometers should be placed close to the centre of mass [34], such as on a harness. However, the placement on top of the shoulder (Figure 1; position 3) limits the collection of information on head movement associated with grazing. For practical on-farm purposes it is likely that accelerometers will likely be included in some form of ear tag. However, several commercial accelerometers may not fit on the ear of young lambs, although advances in miniaturising the technology will undoubtedly change this. The collar placement for accelerometers has been the most commonly used to date, and the results of previous research [14] suggest that, for grazing activity, accelerometer sensors deployed on the ear show similar performance metrics to those on a collar. Similarly, the classification performance of algorithms for walking and standing activities were not different from accelerometry data collected from placement on the ear or a neck collar [19]. Hence, placement on collars appears to be a suitable candidate position for continuing research purposes, in the absence of suitably sized accelerometers for ear deployments. In addition, attaching the accelerometer to a neck collar will capture some head movement but with limited information from minor head movements. Moreover, previous studies in sheep have seldom examined the degree to which classifiers developed in one placement are robust across different placement methods [14,18,19,33], and it remains unclear whether a general classifier can be developed to infer activities for other body positions. As a secondary objective, we sought to investigate the ability of a classifier generated in the collar position to infer similar activity budgets in two other placements.

The aim of the current study was to build a prediction model to classify the diel activity of Perendale sheep at pasture fitted with a neck collar mounting the Actigraph wGT3X-BT^®^ tri-axial accelerometer. This study tested two hypotheses: (1) that raw, untransformed acceleration data could be used to create an accurate classification model capable of inferring diel activity patterns of sheep; (2) that the classification model developed from accelerometers attached to neck collars can be applied to infer activity from accelerometers place on head halters and on body harnesses.

## 2. Materials and Methods

The study was conducted at the Massey University sheep unit, Palmerston North, New Zealand (40°23′28″ S 175°36′21″ E 40 m elevation) between 24 July and 9 August 2018, which is during winter at this location. Ethical approval to conduct this study was obtained from the Massey University Animal Ethics Committee (Protocol No. 16/134, 23 December 2016).

### 2.1. Study Animals and Design

The study comprised two phases: a classification model development phase (P1) and a second phase (P2) to test model performance for different body locations of the accelerometers in identifying the diurnal activity of sheep, with the collar as the reference. P1 was conducted with six lambs that were part of a mob of 27 Perendale ewe-lambs grazing together. The lambs were approximately one year of age and had a mean liveweight of 43 kg (SD = 4.5 kg). For P2, 10 ewe lambs from the same cohort were used, but different individuals than those used in P1. All animals received a standard combination clostridial and leptospirosis vaccine (Ultravac^®^ 7 in 1, Zoetis New Zealand Inc., 8 Mahuhu Crescent, Auckland CBD, Auckland 1010, New Zealand) as well as topical insecticide (Clik^®^, Elanco Animal Health New Zealand Ltd., 106 Wiri Station Road, Wiri, Auckland 2104, New Zealand) to prevent fly strike. They were treated with anthelmintics one and four weeks prior to the study for P1 and P2 respectively and all lambs were determined to be clinically healthy upon physical examination. To allow visual identification in the paddock from a distance, each ewe lamb was coat-sprayed visibly with a unique colour and number on the hind quarter and lateral sides using scourable spray-mark (SprayLine^®^, Midvale, WA 6056, Australia).

### 2.2. Accelerometer

For P1, individuals were fitted with ActiGraph wGT3X-BT^®^ accelerometers (ActiGraph, LLC, Pensacola, FL, USA), which weighed 19 g and were 46 × 33 × 15 mm in size. The wGT3X-BT^®^ records accelerations from the individual’s amplitude (g) and frequency (Hz) of movement across three axes (X for front-to-back; Y for side-to-side; and Z for up-down), and was attached onto the top side of a neck collar with a cable tie, and in P2 two additional monitors were used. One was fastened to a head halter adjacent to the cheek and the second on a ram mating harness being positioned over the shoulders (Figure 1).

The accelerometers were pre-scheduled to sample data at a rate of 30 Hz, i.e., 30 data points per second. For comparison in P2, the accelerometers on the shoulders (body harness) had the front-to-back orientation on the Y axis, side-to-side movement on the X axis and up-down on the Z axis, with these body axes were 90° different to those on the neck collar. For the head halter the accelerometers had employed front-to-back movements on the X axis, side-to-side on the Z axis, and up-down on the Y axis. This difference in orientation was corrected and accounted for prior to analysis.

### 2.3. Behavioural Activity Ethogram

Four categories of behavioural activities were defined a priori based on previous work [6,14] in order to compare behavioural categories collected from accelerometry against behavioural observations. These categories included:

1. Grazing—head down while standing still or slowly moving forward whilst ingesting grass with the muzzle close to the ground.
2. Standing—standing with head up >5 s, minimal head movement (left to right).
3. Walking—head up whilst walking at a slow pace/running at a fast pace. Head raised at or above horizontal plain and eyes open (to include scanning).
4. Lying—lying down with minimal head movement.
5. Other—including scratching, playing etc.

### 2.4. Data Recording and Management

For each phase of the study, raw acceleration data, continuously recorded at 30 Hz, were integrated into five second epochs across the X, Y and Z axes at each of three body positions per sheep.

#### 2.4.1. Model Classification Phase (P1)

Three experimental tests (ET) were conducted. Lambs were in a grazing paddock (ET One), a holding pen (ET Two) or walked through a lane way (ET Three). Each ET was designed to capture a target activity, with grazing, standing, and walking corresponding to ETs One, Two and Three, respectively. All six lambs were filmed during these experimental tests. Video recordings were made using a Samsung NX300 digital camera (Samsung Electronics America, Inc.). All observations were conducted during daylight hours. The starting times for the six observation sessions per day are shown in Table 1. Video observations were taken from a 100 to 200 m distance using the camera’s zoom lens in order to avoid disturbance of the sheep. Although experiment One was designed to capture grazing activity, all other activities were also captured during this time. Lying activity was opportunistically targeted during the late morning period. A mean (SD) of 3.30 h (0.30) of video was recorded for each individual, generating a total video time of ~20 h 40 min (Table 1). Using the behaviours defined in Section 2.3, all videos were watched and coded by the same observer (SJI). An activity profile of each animal was created from videos by annotating and coding activity type at five seconds interval (i.e., five seconds epochs) using CowLog^®^, an open-source software for coding behaviours from digital video [35].

#### 2.4.2. Within-Observer Reliability Test

This test measures the extent to which a single observer obtains consistent results when repeatedly measuring the same behaviour [36]. In this part, the intra-observer agreement was tested using the Kappa statistic by calculating the level of agreement of activity annotations using a subset of 15 min per activity category in four study animals and compared to annotations for activity of the same animals during the initial activity coding. There was a time interval of 18 months between the first and second activity coding. The percentage of exact agreement between the first and second coding of the same behaviour by the observer was calculated, and the within-observer variability was assessed using an intra-class confusion matrix and kappa coefficients (k) [37]. Kappa results were interpreted according to Fleiss et al. [38], where values >0.75 suggested excellent, 0.4 to 0.75 indicated fair-good and <0.4 indicated poor levels of agreement.

#### 2.4.3. Collection of Accelerometer Data from Different Body Locations (P2)

The sensor data from each body location were collated for a 72-h period commencing at 0900 h on the day of attachment of the sensors to the lambs (i.e., Tuesday), and presented as three daily blocks, that is per 24 h. As mentioned above, the orientation of the X, Y and Z axes differed between the attachment methods. This was adjusted for prior to analysis. Then the classification model was applied to deduce activity types at each 5 s interval. We then separately compared the activity budgets for the head halter and the harness to the activity budget of the collar.

### 2.5. Statistical Analysis

All data computation and statistical analysis was conducted in R version 3.5.2 [39].

#### 2.5.1. Descriptive Statistics

The frequency of occurrence of the coded activity were described and two-dimensional plots were used to describe the relationship between activity types and the X-Y, X-Z and Y-Z axes.

#### 2.5.2. Phase One—Building Classifier Model

Activities classified as ‘other’ were removed. A random forest algorithm (R package ‘randomForest’, [40]) was used to develop an activity classification model using the raw X, Y and Z accelerometer data to predict the activity types observed in the labelled dataset, running 1000 iterations. This method implements out-of-bag error estimation for robust and unbiased inferences. In each iteration, the algorithm randomly samples data points and variables, and then combines the output at the end. The output of the out-of-bag random forest model (hereafter called classifier) was then used to predict behaviours using the entire labelled dataset, and model predictions were compared to the gold standard (video observations) to compute a confusion matrix to evaluate the performances of the classifier. Two metrics were used for overall classifier performance across all activities: the overall accuracy and overall misclassification rate. To evaluate the performance of the classifier for each individual activity type separately, four performance metrics were calculated as outlined in Equations (1)–(4):

(1) Sensitivity=TP/(TP+FN) 

(2) Specificity=TN/(TN+FN)

(3) Precision=TP/(TP+FP)

(4) Accuracy=(TP+TN)/(TP+TN+FP+FN) 

where, TP (true positive) corresponds to the number of epochs where the behaviour of interest was correctly predicted by the classifier. TN (true negative) is the number of epochs where the behaviour of interest was correctly classified as not having occurred. FN (false negative) is the number of epochs where the behaviour of interest was observed but not inferred by the classifier. FP (false positive) is the number of epochs where the behaviour of interest was inferred by the classifier but not observed. To further validate the predictive ability of the classifier model, a “leave-one-out” cross-validation was used based on individual sheep removal, as the observations were clustered by individual. Data points for each individual lamb were removed sequentially from the labelled dataset, the model was trained using the remaining five lambs and validated on the lamb removed.

#### 2.5.3. Phase Two

For P2, the daily activity budgets (proportion of time spent grazing, resting, and walking) were calculated for each ewe lamb (*n* = 10) and compared between accelerometer placements. The daily proportions of each activity were summarised as mean per hour of day and described for each placement of the sensor on the animal’s body.

A Dirichlet regression with log link was then fitted to model the relative hourly proportion of time spent in each activity for each sheep, as a function of the accelerometer placement and the day. As the multivariate generalization of the beta distribution, the Dirichlet distribution accounts for the numerical constraint associated with compositional data such as activity budgets, whose components sum to 1 [41], and allows for the simultaneous assessment of the effects of covariates on the relative contribution of multiple activities [42]. The R package ‘DirichletReg’ [43] was used to model the response variable activity budget (hourly proportion of performing the activities) and the explanatory variables were position (head halter and body halter, reference = neck collar) and day (the trial was run for 3 consecutive days).

To endeavour to quantify sheep activity occurring during daylight and those occurring at night, the collar-derived accelerometry daily data were divided into the mean hourly sunset to sunrise times over the three days of activity monitoring (i.e., from 07.30 to 17.18 h for daylight hours; www.timeanddate.com, 2018, accessed 23 March 2019).

## 3. Results

### 3.1. Descriptive Statistics for Labelled Dataset and 2-D Plots (Phase 1)

**Figure 2:**
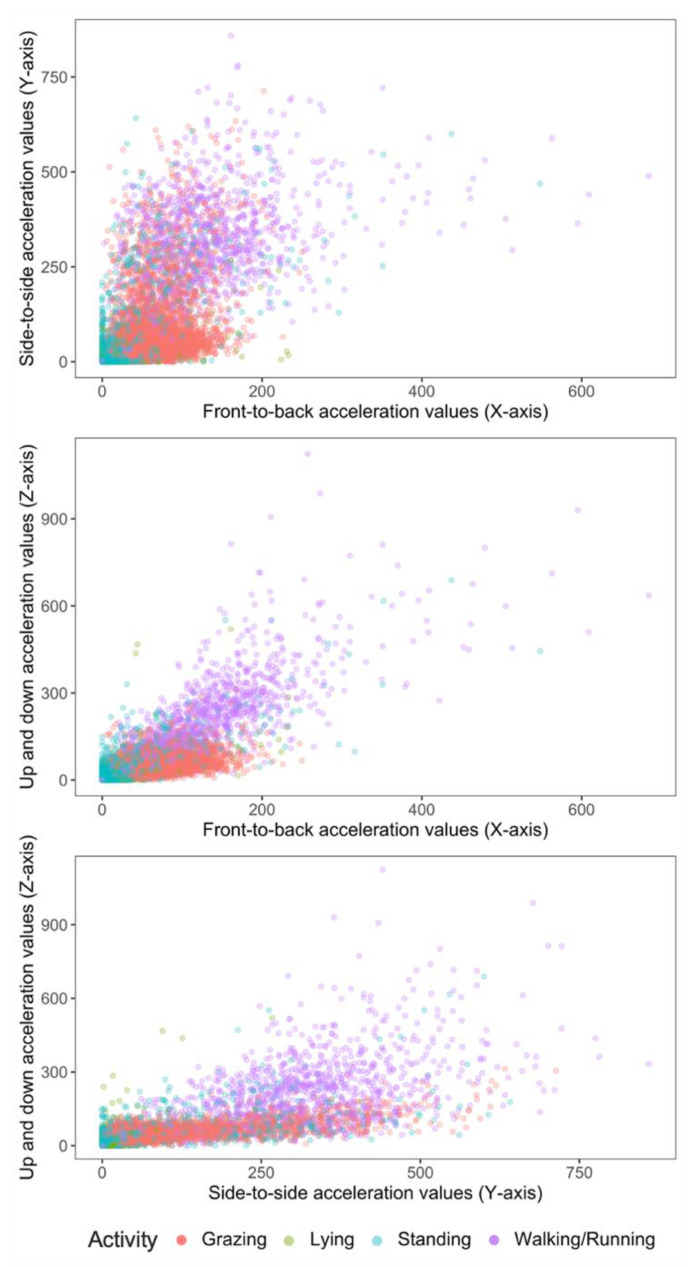
2-D scatter plots of acceleration signals recorded in X, Y and Z axes of six ewe lambs during periods of grazing, standing, lying and walking.

The epoch counts (total time) labelled for activities were grazing—4855 (6 h 45 min), lying—2573 (3 h 34 min), standing—2639 (3 h 39 min), and walking/running—1078 (1 h 30 min). The activity budget is shown in Table 2. Grazing was the most frequent activity. Activity data are described in two-dimensional plots (Figure 2) between each of the predictor axis, i.e., X-Y, X-Z and Y-Z axes, where X, Y and Z axes correspond to axis1, axis2 and axis3, respectively.

### 3.2. Within-Observer Agreement Test

A summary of the activity data recorded by one observer on two occasions (18 months apart) is shown in Table 3. The overall accuracy between the first and second observations was 99%. Walking and standing behaviours were the most frequent to be misclassified, but with a misclassification error ≤4% between the activities. The Kappa value (k = 0.98) suggests that there was excellent agreement between the first and second coding of the same observer.

### 3.3. Random Forest Model

**Figure 3:**
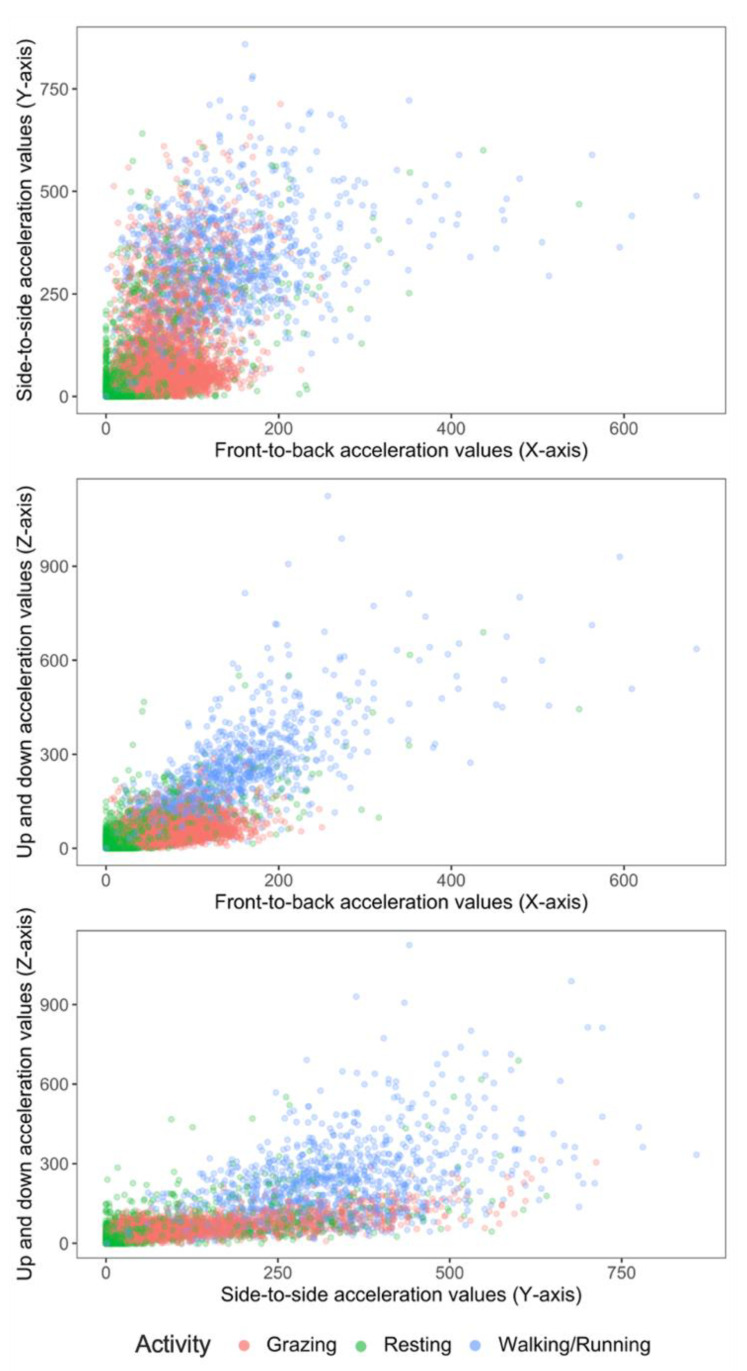
2-D scatter plots of acceleration signals recorded in X, Y and Z axes of six ewe lambs during periods of grazing, resting (standing or lying activity) and walking.

The best out-of-bag classification model (classifier) was derived by combining the standing and lying behaviours into one behaviour (=resting). This improved model prediction and reduced misclassification by 15%. Two-dimensional scatter plots of grazing, walking and the newly categorized resting extracted from the accelerometers mounted on neck collars using raw X, Y and Z acceleration values are shown in Figure 3.

The confusion matrix of the final model predictions against the observed activities (video recorded) is shown in Table 4.

The performance metrics calculated from the classifier model for each activity is shown in Table 5.

### 3.4. Leave-One-Out Cross Validation by Individual Sheep Removal

The overall accuracy for each round of prediction was 88% (Kappa 0.8; 95% CI 87 to 90), 87% (Kappa 0.8; 95% CI 85 to 88), 88% (Kappa 0.8; 95% CI 87 to 90), 88% (Kappa 0.8; 95% CI 86 to 89), 92% (Kappa 0.9; 95% CI 90 to 93) and 87% (Kappa 0.9; 95% CI 90 to 93), respectively. This resulted in a mean model prediction accuracy of 88% (SD = 1.7%). The performance of random forest models analysed at the level of the individual ewe-lambs (i.e., trained by five individuals’ labelled datasets to predict the sixth individual) is shown in Table 6.

### 3.5. Testing Classifier Model Performance to Estimate Sheep Diel Activity and at Alternative Body Positions (Phase 2)

The daily activity budgets deduced (proportion of time spent grazing, resting, and walking) using the classifier developed in Phase 1 from the accelerometer data obtained by fitting the accelerometer to a collar (neck, as reference), a halter (head) and a harness (body) are presented in Table 7.

At the collar reference position, grazing was the primary activity of sheep for all three days of monitoring during daylight hours (Table 8). Grazing time during daylight hours represented approximately 82% of the total grazing time.

**Figure 4:**
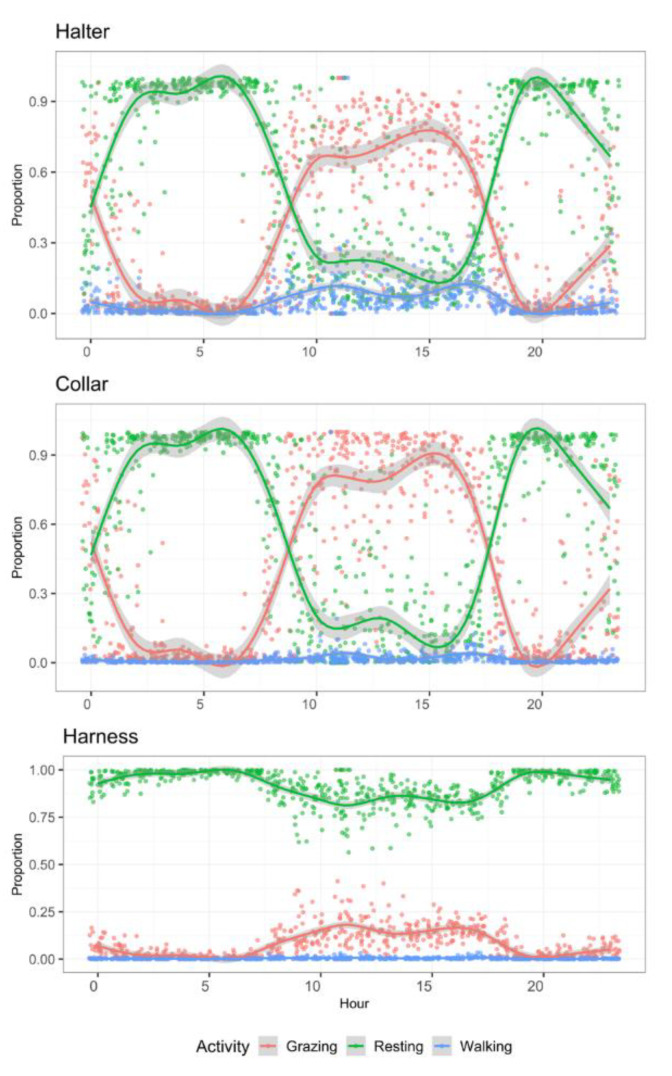
Mean daily activity budgets of ewe lambs (*n* = 10) per hour of day allocated to three activities monitored for 72 h with tri-axial accelerometers at three positions on the animal’s body.

Collars and halters produced similar proportions of time spent in each activity type (Figure 4). However, results derived from harness accelerometers were different for all three activity types. Resting was strongly overestimated, whereas the other two types were strongly underestimated.

Results of the multivariable Dirichlet model to compare accelerometer body placements are presented in Table 9. They indicate that predictions of grazing and resting activities were not statistically different using an accelerometer on a head halter, compared to the neck collar. On the other hand, the daily proportion of time spent walking was overestimated by sensors placed on the head halter, as compared to the collar. At the body harness placed sensors, the daily proportion of time spent grazing was underestimated, while time spent resting was overestimated. Only the total walking time did not differ between harness and collar derived data.

## 4. Discussion

Results from this study show the utility of tri-axial accelerometers in capturing the diel activity of sheep on pasture. Acceleration data were used to identify three behavioural activity classes performed by ewe lambs. The random forest classification algorithm (classifier) had an overall accuracy of ~90% when related to video footage that recorded the activity at the same time. This study shows that raw X, Y and Z acceleration data can be used to develop classification algorithms for the grazing, resting and walking activities of Romney sheep on pasture. This algorithm was developed without transforming the data to create the summary features. Traditionally, X, Y and Z axis values are used to calculate summary features, which are then evaluated and subsequently tested for accuracy of prediction of activity [4,6,33,44,45,46], although the number of features generally differ across studies. Since random forests allow for non-linear relationships between the covariates and the outcome (behaviour class membership probability) to be detected [47,48], they proved satisfactory for the purposes of this present study. Furthermore, the classifier developed was extended to accelerometer data at two other placement positions to infer the activity budgets of lambs in comparison to the collar position where it was developed, which is discussed below.

Grazing activity had the lowest misclassification rate (6%) among all activity types predicted by the classifier. With a classification precision of 86%, grazing activity was, however, 10% less precisely predicted than resting, but 10% more precisely predicted than walking. This is likely due to grazing having a higher rate of false positives than resting. Walking was misclassified as grazing 24% of the time by the classification model developed from the collar position, which may be attributable to the fact that sheep often quickly alternate between moving with head up and grazing with head down [49]. It is plausible that this difficulty in differentiating these two activity types with higher resolutions relates to the sampling frequency. In contrast, Barwick et al. [14] found a high prediction accuracy for walking behaviour, readily differentiating walking events from grazing and standing behaviours. This may have been achieved as a result of the transformation of the raw data in their study. In the present study, although walking was only predicted correctly 72% of the time, it was considered satisfactory to maintain it as a predicted category. Similar results to those of the present study were shown for estimations of walking activity by accelerometers placed at the collar location by Walton et al. [19]. Also, it was the intention of the authors to predict grazing behaviour apart from other activities, hence not combining with walking activity to create an active category [14].

Conversely, to improve the accuracy of the final algorithm, standing and lying were combined into a resting category. The acceleration signals of standing and lying were similar, as seen in Figure 2 and Figure 3. This would explain the misclassification between both categories and hence warrant combining the categories. This recategorization of standing and lying into resting improved the accuracy and precision of the classification to 93 and 96%, respectively. Barwick et al. [14] proposed further studies employ this approach and merge the classifications of lying and standing behaviour into an “inactive” behaviour category, as no clear grouping was evident for these activities in their study. It is possible to speculate that the X, Y and Z signals only differ at the collar position when animals are either transitioning between lying down and standing up, but once they stand or are lying the X, Y and Z signal is the same. This activity transition period can be the target of future efforts at building classifiers, as it should be pointed out that the different activities indicative of inactive or resting states could also be important in on-farm management as indicators of health issues.

When comparing two other accelerometer placement positions to the collar-derived data, at least one activity frequency was not different. One way to look at this is that the classifier developed at the collar position was robust enough to capture activity budgets at other accelerometer placement positions. However, care in interpreting this finding is warranted because the other placement positions were not compared to the video observations in this study. Other studies investigating placement methods develop classifiers for each placement method and hence compared sensor data at these positions to video data [13,14,19]. In the current study, the results from using a single classifier developed from one placement method to infer activity at another suggests that little activity may have been recorded at the harness position. Hence, resting was overpredicted, with grazing activity underpredicted using this attachment method. It is also likely that a significant difference between collars and harnesses for walking activity may have been seen because walking was a rarely predicted activity. Since comparisons were made for the time budgets and not the individual classification of the activities, it is conceivable that the harness-placed accelerometers may have recorded walking activity at a very different time than the collar. However, when grazing is the target activity, it is unlikely that accelerometer technology located on a body harness would reliably detect grazing activity. Future studies can seek to corroborate acceleration signatures from these different placement methods with time stamps of those occurring activity and then assess the performance of a classifier derived in one position to the others, and vice versa. In all, this finding highlights the challenge of applying classifiers developed from tri-axial accelerometer data from one placement to another placement, not the least applying classifiers developed from other species.

In terms of quantifying the diel activity of sheep, the collar mounted sensor showed that the ewe lambs followed a daily pattern of activity consistent with previous observations [50,51], indicating more grazing activity in day light hours, with resting predominating between sunset and dawn hours. The average daily time budget of the ewe lambs by hour of day showed that their grazing activity started to rise just after 06.00 h, and peaked shortly after 15.00 h, before starting to decrease (Figure 4), in accord with the observations of Bueno and Ruckebusch [51]. Generally, sheep graze from sunrise to dusk, stopping sporadically to chew their cud (which is a resting activity despite small head movements) and this grazing period can be up to seven hours of the day [50], but a range of 7 h to 12 h a day has been reported for sheep daily grazing time [52]. This grazing activity pattern is typical of sheep in Mediterranean climes [50], although in hot arid environments, sheep were shown to graze with less intensity as temperatures rose from 10.00 h to 15.00 h, with grazing steadily rising again from 16.00 h [53]. Sunset hours were associated with 14% (95% CI 8% to 20%) of total grazing time. Mohammed et al. [54] comparably noted that the time herbivores spent grazing at night varied between 10 to 30% of total grazing time. Elsewhere, sheep that were housed in pens at night were found to spend 72% of total daylight activity grazing [54]. As sheep have been shown to compensate for their daily grazing by grazing longer in daylight when they do not have access to pasture at night [55], this may account for the higher proportion of time spent grazing by Mohammed et al. [54] compared to the 64% sheep spent in the present study. We further suggest that our algorithm included rumination activities into the resting category, which might be why the ewe lambs were estimated to rest for upward of five hours on average, compared to the 3 to 4 h sheep were reported to spend sleeping by Arnold [50].

Overall, it is possible to apply this output in a variety of contexts. For example, infection with gastrointestinal nematodes is known to affect voluntary feed intake [56,57] which is presumably reflected in grazing activity. Hence, grazing behaviour derived from accelerometer data could be used as a proxy for feed intake, and hence as an early warning sign for nematode infection [27]. Head halters on cows that measured jaw movement combined with pedometers showed that dairy cows grazed for longer whilst benefiting from the persistent activity of anthelmintics whilst untreated cows ate less and presumably had to divert resources to mount an immune response against ingested larvae [58]. The results in the current study can be used to infer similar changes in sheep. Overall activity data calculated from the Actigraph tri-axial accelerometer allowed the detection of reduced total activity in parasitized young sheep using dynamic vectorial body acceleration (VeDBA; [27]). The development of the classifier model in the present study can now allow the optimal placement method for further differentiation of activities and how activities change in response to the effects of parasites on their hosts’ welfare and health. These results could also be used to study other food intake disorders, such as rumen atony, rumen tympany, and secondary inhibition of the gastrointestinal motility resulting from diseases with severe pain.

## 5. Conclusions

This study demonstrates the value of raw, untransformed accelerometry data to predict discrete numerical signatures associated with grazing, resting and walking activity of sheep. The change in activity budgets in relation to time of day derived from sensor data were similar to those reported in other studies using direct observation, showing that sheep spend most of their daytime grazing. This research confirms that tri-axial accelerometer sensors can be a very effective tool for identifying the grazing activity of sheep, but a classifier developed in one attachment location may not be robust to infer activity at different attachment locations. These findings should facilitate further research for identifying day and night-time activity in sheep on pasture in response to changing internal and external environmental conditions. Overall, the technology shows promise to inform early identification of deviations in normal diel activity of sheep and provide managers with decision support towards better health and welfare outcomes.

## Figures and Tables

**Table 1:** Starting times and duration of focal behaviour observation sessions recorded by video across three experimental periods on ewe lambs (*n* = 6) fitted with a collar mounting an Actigraph^®^ wGT3X-BT accelerometer sensor.

Date	Focal Behaviour	Start	End	Duration (mins)
2/08/2018	Grazing	14:09:40	14:39:30	29:59
2/08/2018	Grazing	14:41:05	14:51:00	17:05
3/08/2018	Lying	11:15:00	11:45:55	31:00
9/08/2018	Standing	10:46:50	11:16:35	29:59
9/08/2018	Standing	11:17:15	11:22:15	05:09
9/08/2018	Walking	11:28:00	11:44:55	17:03
11/08/2018	Grazing	13:17:35	13:47:25	29:59
11/08/2018	Grazing	13:48:40	14:18:30	29:59
11/08/2018	Grazing	14:19:10	14:34:10	15:40

**Table 2:** Activity budget percentage (95% confidence interval) of overall epochs (5 s periods) that were classified as grazing, lying, standing, walking/running or other from filmed ewe lambs.

Activity	Percentage (95% CI)
Grazing	38.8% (38.0 to 39.6)
Lying	20.6% (19.9 to 21.3)
Standing	21.1% (20.4 to 21.8)
Walking/Running	8.6% (8.1 to 9.1)
Other	11% (10.5 to 11.5)

**Table 3:** Count of activity for four ewe lambs at five seconds interval over a one-hour period. Observations coded by one observer on two occasions (18 months apart).

	1st Coding				
**2nd Coding**	**Grazing**	**Lying**	**Standing**	**Walking/Running**	**Total**
Grazing	173	0	0	0	173
Lying	0	177	0	0	177
Standing	0	0	150	6	156
Walking/Running	0	0	3	133	136
Total	173	177	153	139	642

**Table 4:** Confusion matrix of the best classifier using random forest, showing the predicted activity for neck collar-mounted accelerometers recording activity of six sheep during four sampling days (5 s epochs). Values across the diagonal (bold) represent those activities that were correctly identified, true positives. Values in the matrix are the number of epochs.

Predicted Activity			
**Observed Activity**	**Grazing**	**Resting**	**Walking**
Grazing	**4562**	154	139
Resting	459	**4650**	103
Walking	256	50	**772**

Out-of-bag misclassification rate: 10.4%.

**Table 5:** Performance metrics of random forest classifier algorithm for grazing, resting, and walking activities of ewe lambs.

Activity	Accuracy	Precision	Specificity	Sensitivity
Grazing	91%	86%	88%	94%
Resting	93%	96%	96%	89%
Walking	95%	76%	97%	72%

**Table 6:** Performance of the random forest classification algorithm predictions across individual ewe lambs (*n* = 6). Data shown for each lamb when data for other 5 lambs were used to develop the algorithm.

Lamb		Grazing				Resting				Walking			
	*n*	Acc.	Prec.	Spec.	Sens.	Acc.	Prec.	Spec.	Sens.	Acc.	Prec.	Spec.	Sens.
1	1838	89%	80%	86%	94%	93%	95%	96%	89%	94%	80%	99%	50%
2	1853	88%	85%	93%	80%	90%	89%	87%	93%	94%	74%	97%	73%
3	2146	90%	89%	89%	91%	93%	91%	93%	91%	93%	63%	97%	52%
4	1717	89%	83%	87%	92%	91%	94%	94%	88%	95%	83%	98%	69%
5	2887	93%	90%	91%	96%	93%	97%	97%	88%	97%	81%	98%	88%
6	1504	89%	89%	84%	92%	91%	95%	98%	76%	93%	64%	94%	87%

*n* = number of predictions made for individual lamb; Acc. = Accuracy; Prec. = Precision; Spec. = Specificity; Sens. = Sensitivity.

**Table 7:** The mean (95% confidence limits, CL) daily time spent grazing, resting, and walking for three days recorded continuously by tri-axial accelerometers positioned on a neck collar, head halter and body harness of ewe lambs (*n* = 10).

Activity	Position		
	Collar (95% CL)	Halter (95% CL)	Harness (95% CL)
Grazing	39% (31, 48)	35% (28, 42)	7.8% (6, 10)
Resting	59% (51, 67)	60% (52, 68)	91.9% (90, 93)
Walking	2% (1, 2)	5% (4, 6)	0.3% (0.2, 0.4)

**Table 8:** Activity time budgets of grazing sheep during 11 h of daytime and 13 h of sunset hours derived from neck collar mounted accelerometers on 10 ewe lambs over three monitoring days.

	Mean	95% CI
**Total daytime hours**		
Grazing	64%	55% to 74%
Resting	33%	23% to 43%
Walking	2%	2% to 3%
**Total sunset hours**		
Grazing	14%	8% to 20%
Resting	85%	80% to 91%
Walking	1%	0% to 1%

**Table 9:** Result of the Dirichlet regression model of the relative allocation of daily activities (grazing, resting, walking/running) for 10 sheep over 3 monitoring days. Reference category for the model is the neck collar.

	Estimate	SE	*p*
**Grazing**			
(Intercept)	−1.07	0.21	<0.001
Position (Halter)	−0.29	0.22	0.174
Position (Harness)	−0.48	0.23	0.034
Day	1.71	0.08	<0.001
**Resting**			
(Intercept)	−1.11	0.18	<0.001
Position (Halter)	−0.09	0.22	0.671
Position (Harness)	1.53	0.23	<0.001
Day	1.86	0.07	<0.001
**Walking**			
(Intercept)	−2.36	0.23	<0.001
Position (Halter)	0.75	0.22	0.001
Position (Harness)	0.08	0.22	0.731
Day	1.16	0.09	<0.001

## Data Availability

Data available upon request.
